# Adaptable Microporous Hydrogels of Propagating NGF‐Gradient by Injectable Building Blocks for Accelerated Axonal Outgrowth

**DOI:** 10.1002/advs.201900520

**Published:** 2019-07-11

**Authors:** Ru‐Siou Hsu, Pei‐Yueh Chen, Jen‐Hung Fang, You‐Yin Chen, Chien‐Wen Chang, Yu‐Jen Lu, Shang‐Hsiu Hu

**Affiliations:** ^1^ Department of Biomedical Engineering and Environmental Science National Tsing Hua University Hsinchu 30013 Taiwan; ^2^ Department of Biomedical Engineering National Yang Ming University Taipei 11221 Taiwan; ^3^ Department of Neurosurgery Chang Gung Memorial Hospital Linkou Medical Center and College of Medicine Chang Gung University Taoyuan 33305 Taiwan

**Keywords:** adaptable hydrogels, controlled release, injectable, porous scaffolds, tissue regeneration

## Abstract

Injectable hydrogels in regeneration medicine can potentially mimic hierarchical natural living tissue and fill complexly shaped defects with minimally invasive implantation procedures. To achieve this goal, however, the versatile hydrogels that usually possess the nonporous structure and uncontrollable spatial agent release must overcome the difficulties in low cell‐penetrative rates of tissue regeneration. In this study, an adaptable microporous hydrogel (AMH) composed of microsized building blocks with opposite charges serves as an injectable matrix with interconnected pores and propagates gradient growth factor for spontaneous assembly into a complex shape in real time. By embedding gradient concentrations of growth factors into the building blocks, the propagated gradient of the nerve growth factor, integrated to the cell‐penetrative connected pores constructed by the building blocks in the nerve conduit, effectively promotes cell migration and induces dramatic bridging effects on peripheral nerve defects, achieving axon outgrowth of up to 4.7 mm and twofold axon fiber intensity in 4 days in vivo. Such AMHs with intrinsic properties of tunable mechanical properties, gradient propagation of biocues and effective induction of cell migration are potentially able to overcome the limitations of hydrogel‐mediated tissue regeneration in general and can possibly be used in clinical applications.

## 1 Introduction

Injectable materials with highly integrated functionalities have considerable benefits for regenerative medicine owing to their potential ability to mimic hierarchical natural living tissue and guide tissue regeneration with minimally invasive implantation procedures.^1^, ^2^ Because of their tunable properties and ability to transport cargo, the materials can fill complex shaped defects, regulate cell behavior and guide cell growth, as well as the less invasive delivery procedure.^3^ Hydrogel, as a scaffold, one of the most attractive injectable materials, displays amenable elasticity and enables facile diffusion of biomolecules or therapeutic agents for constructing a 3D extracellular matrix (ECM).^4^, ^5^, ^6^ Some hydrogels have been approved for clinical uses in tissue engineering, such as in autologous fat, bovine collagen, and avian‐derived Hyaluronic acid.^7^, ^8^ Others have also been applied in ex vivo 3D living tissues, and disease modeling.^9^, ^10^, ^11^

Despite recent advances in tissue engineering, challenges of injectable hydrogel still exist.^12^ First, the limitations of injectable nonporous hydrogel are weak cellular permeability and do not precisely match the rate of material degradation to tissue development for cellular infiltration, proliferation, and vascularization.^13^ Degradation of materials by hydrolytic and enzymatic mechanisms can be manipulated through the molecule design and crosslinking density. However, an imprecise match of the material degradation rate to the rate of tissue development leads to insufficient scaffolding or the promotion of fibrosis.^14^, ^15^, ^16^ Decreased mechanical durability of materials caused by a decoupling of cellular infiltration is also unavoidable. Second, propagating the gradient of growth factor levels and directing cells with a highly organized ensemble in vivo for injectable hydrogels are still difficult. In addition, regenerative deceleration can be caused by the inappropriate mimicking by the hydrogel of the elastic and mechanical properties of native tissue for orchestrating cells.^17^, ^18^ Thus, there is a strong need to develop a functionally and gradient spatial structurally optimized injectable hydrogel scaffold with tunable mechanical properties,^19^, ^20^ and efficient to promote the acceleration of tissue growth for applications in biological research.

A simple solution to these tissue regeneration problems is to engineer formulations of hydrogels with a suitable porosity and interface to instruct tissue formation.^21^, ^22^, ^23^, ^24^ Porous scaffolds that serve as tissue regeneration templates can guide new tissue before degradation, yet most of these scaffolds are noninjectable and exhibit poorly interlinked pores. Toward this end, injectable building blocks with connecting pores have been applied routinely. These building blocks possess specific properties such as injectability, self‐healing, interconnected pores, physical and chemical crosslinking that can be tailored by controlling their external molecular forces and physicochemical properties.^25^, ^26^ For example, Diba et al. reported highly elastic and self‐healing composite colloidal gel assembled from pH‐controlled oppositely charged nanosized particles.^27^ However, the pores of nanosized building blocks are too small to guide cell proliferation.^29^, ^30^ By controlling the components of microgels, the resulting dynamic and adaptive gel network serves as a promising strategy for bone and organ regeneration.^31^ For example, Segura and co‐workers reported that the assembled injectable microporous gel facilitates cell infiltration and accelerates wound repair processes at the brain‐damaged cavity.^1^, ^3^ Relevant applications unique to microporous gels also include distinct physiological niches, such as cardiac, skin, and neural niches. The development of annealed gel creates an opportunity for tissue engineering.

Even with the current breakthroughs in injectable gels, interconnected porous scaffolds have found limited applications in nerve regeneration, e.g., peripheral nerve injury (PNI),^32^, ^33^ due to the potential properties such as low gel formation rates, weak propagation gradient of growth factors, and uncontrollable inter/intramolecular modulus. For rapid gel formation, adaptable hydrogels provide adaptable linkages that can be immediately broken and reformed in a reversible manner without external triggers, facilitating filling and fixing in the nerve conduit during surgery. Furthermore, the key problem of peripheral nerve regeneration is accelerating Schwann cell (SC) migration across the injury site to achieve the effective formation of bands of Büngner in a short time and directing of motor and sensory peripheral axon outgrowth at the intrinsic rate.^34^, ^35^ In this regard, Lee et al. designed a biocompatible axonal guidance device and the in vitro study showed that a propagating gradient of IGF‐1 directs axonal outgrowth of up to 5 mm to promote axonal growth, indicating the importance of gradients.^36^ However, a complex PNI with a certain axonal loss and sizable gap defect exhibits a slowly regenerative rate of the defected nerve and delays functional recovery.^37^ Therefore, engineering a material that can provide directional axonal growth while maintaining SCs may be essential for rebuilding the electrical and chemical signals between the distal and proximal stumps after PNI.

**Figure 1:**
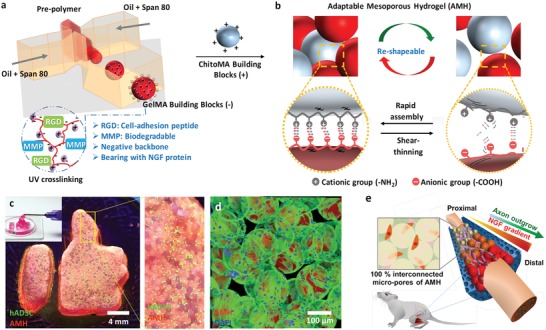
Microfluidic fabrication of adaptable microporous hydrogels (AMHs) for the creation of injectable scaffolds with 100% interconnected micropores. a) Schematic illustration of monodispersed gelatin methacrylamide (GelMA) building block formation using a microfluidic water‐in‐oil emulsion system. GelMA is crosslinked through a photo‐activated radical initiator and UV light. b) Micro‐network scaffold is formed by mixing oppositely charged building blocks via adaptable interaction. c) AMHs are injectable and moldable to form complex and macroscale shapes through physical crosslinking. AMH can be performed in the presence of live human adipose‐derived stem cells (hADSCs). d) Fluorescence image of a cell‐laden micronetwork scaffold. e) Schematic illustration of AMH with a propagating NGF‐gradient for directed and accelerated axonal outgrowth in vivo.

In this study, we propose an adaptable microporous hydrogel (AMH) to accelerate and direct peripheral nerves based on a unique type of microsized building block that spontaneously forms interconnected pores, propagates the gradients of neuron growth factors, tailors the stiffness, and controls the pore sizes in nerve conduits. Through microfluidic fabrication, building blocks are constructed by a bottom‐up synthesis employing photocrosslinkable gelatin methacrylate (GelMA) and chitosan oligmer‐methacrylate (ChitoMA) as negatively and positively charged building blocks, respectively (**Figure** **1**a). GelMA is a photo‐crosslinking hydrogel composed of modified collagen components. With the benefit of denatured collagen, GelMA retains natural cell‐binding motifs, such as cell adhesive peptide (arginyl‐glycyl‐aspartic acid, RGD) as well as matrix metalloprotease peptide (MMP) sequences that allowed cell controlled material degradation and subsequent resorption.^38^ Furthermore, chitosan degradation products have been documented to facilitate peripheral nerve regeneration.^39^ This AMH is reshapable and reassembles through shear‐thinning force and strong cohesive properties (Figure 1b), facilitating the formation of a stable 3D porous scaffold. Such an interconnected injectable porous scaffold with suitable micropores for prompt cell migration as well as offer mechanical support and transports biomolecular cues to manage cell adhesion and growth (Figure 1c,d). The adaptable microporous scaffold constructing cell‐penetrable pore sizes in real time, integrated with a propagating gradient of a NGF in a nerve tube (Figure 1e), directs axon outgrowth of up to 4.7 mm in 4 days in vivo and well aligned axons with functional recovery within 30 days postsurgery. Such synergistic effects of injectable AMHs of rapid bonding, precise pore control, and tunable molecular cue gradient formation effectively create a new horizon for applications in tissue regeneration.

## 2 Results and Discussions

### 2.1 Synthesis and Physicochemical Characterization of AMH

**Figure 2:**
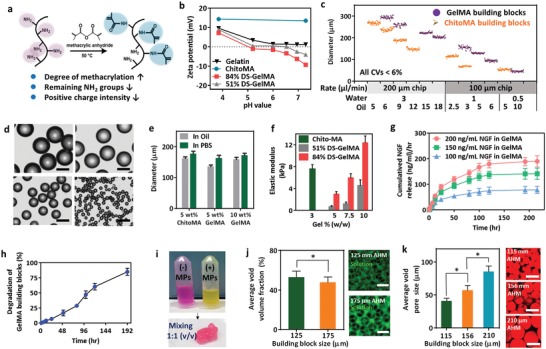
Synthesis and physicochemical characterization of AMHs. a) Chemical reactions for the methacrylation of gelatin via the amino groups of gelatin with methacrylic anhydride. b) The effect of the degree of substitution (DS) on the zeta potentials of GelMA and ChitoMA. c) The operational regime for microfluidic building block generation displayed an order of magnitude in size at various conditions (with CVs < 6%). d) Light microscopic images of building blocks. The scale bar is 200 µm. e) The size distribution of building blocks in paraffin oil and PBS solution. f) The elastic modulus of single building blocks at different DS and concentrations. g) The in vitro protein release from 10 wt% GelMA building blocks with collagenase. h) Degradation profile of 10 wt% GelMA building blocks incubated with collagenase (*n* = 5, mean ± s.d.). i) GelMA and ChitoMA building blocks in the Eppendorf tubes before mixing. The gelation of mixing two types of building blocks with equal volume. j) The average void volume fractions of 125 and 175 µm of building blocks (left). The AMH composed by equal sizes of building blocks was immersed to a fluorescence solution (50 kDa of fluorescent dextran) and the unoccupied volume was filled by the solution (right). 3D reconstructed images revealed the characteristics of pores in the AMH (*n* = 6, mean ± s.d., *p < 0.05, *t*‐test) The scale bar is 200 µm. k) The void pore sizes of 115, 156, and 210 µm of building blocks (*n* = 6, mean ± s.d., *p < 0.05, *t*‐test). The scale bar in fluorescence image is 200 µm.

The GelMA and ChitoMA building blocks were prepared by a robust microfluidic water‐in‐oil (w/o) emulsion approach to precisely segment droplets, which subsequently undergo photopolymerization as previously described (Figure S1, Supporting Information).^40^ To obtain the photopolymerizable building blocks, the amine groups of gelatin were substituted with methacrylate anhydrate (MA) (**Figure** **2**a). Both high and low degree of substitution (DS) of methacrylation of GelMA were fabricated and the DS of GelMA was determined by NMR spectroscopy (high and low DS‐GelMA are 84% and 51%, respectively, (Figure S2 in the Supporting Information)). The zeta potentials of GelMA and ChitoMA exhibited pH‐dependent behavior and possessed oppositely charged at pH 7, indicating the attractive electrostatic interactions in neutral condition (Figure 2b). Due to the presence of amino groups in chitosan, it is a cationic polyelectrolyte (p*K*a ≈ 6.5). After emulsifying and curing on a microfluidic chip, the building blocks fabricated by GelMA and ChitoMA with narrow size distribution can be observed in Figure 2c. Once the building block was redispersed in PBS solution, the structures remained intact and could be stained by various fluorescence dyes with high photostability (Figure S3, Supporting Information).

By simply controlling the microfluidic channel size and the ratio of the aqueous/oil rates, both negatively and positively charged building blocks with an average diameter ranged from 50 to 310 µm with low polydispersity can be formed. Under the same flow rate in an identical chip, the difference in size of ChitoMA and GelMA building block is caused by their different viscosity. Under the optical microscope, the building blocks with narrow size distribution can be also observed in Figure 2d, and these microspheres can be preserved in deionic water for 3 months without any server changes. Figure 2e shows the size changes of building blocks in water and oil, suggesting the slight swelling in water.

The mechanical properties of building blocks can be controlled by adjusting DS and polymer concentration (Figure 2f). The elastic modulus of 5 wt% GelMA with DS of 51% and 84% is 1.2 and 3.6 kPa, respectively. Once the concentration of GelMA was increased to 10 wt%, the modulus of gel can reach to 4.5 and 12 kPa for DS of 51% and 84%. As the literature documented that the mechanical properties of subtract and microenvironment are the crucial issue to regulate the cell adhesion, proliferation, and differentiation.^13^ Concerning repair of axons in the peripheral nerves, the modulus of the matrix (3.80 kPa for SC proliferation) has to fit the SCs to obtain the prevalent in vivo bipolar morphology to connect proximal and distal of nerves.^33^ In our study, the elastic modulus of 10 wt% of GelMA with 51% DS is 4.5 ± 0.3 kPa, matching the preferred stiffness of the SCs.

To investigate the formation of NGF gradient in a conduit, various concentrations of NGF including 200, 150, and 100 ng mL^−1^ were embedded in GelMA building blocks, and then, three types of AMH containing the NGF‐loaded GelMA and empty ChitoMA building blocks at equal volume were placed in a tube one by one to form three sections. The release profiles of each section were monitored under a 0.5 U mL^−1^ collagenase solution. Both the loading efficiency and the amount of NGF release from the AMH were determined using an NGF rat ELISA Kit (Arigo Biolaboratories Corp). Through in situ microfluidic fabrication, the loading efficiency of NGF in the AMH is ≈95%. The stability was attributed by complexation between NGF (pI of 9.3) and GelMA building blocks (pI of ≈5) in a neutral solution.^41^ Without adding the collagenase to NGF‐loaded AMH, the sustained release of NGF reaches about 20% within 1 week.

While applying the collagenase, the obvious NGF release from AMH was observed, indicating that the degradation of AMH was the main factor to cause NGF release. Figure 2g exhibits three similar release patterns of NGF from building blocks but demonstrated the significant differences in release amounts under collagenase solution. While loading higher concentration of NGF in AMH, the stronger signal of NGF could be monitored in each time point. For example, after 100 min, the cumulative release amount of 200 ng mL^−1^ NGF embedded in AMH was threefold greater than that in 100 ng mL^−1^ NGF embedded in AMH. The cumulative release was linear dependence of the square root of time, revealing that the degradation and concentration gradients possibly dominated the release pattern based on the Fick's second law (Figure S4a, Supporting Information). Furthermore, with the assistance of collagenase, the degradation is less than 5% for 24 h, and the linear degradation rate can be observed in the following 196 h. After 8 days, the percentage of degradation reached 80% (Figure 2h). The surface erosion and degradation of AMH actuated by collagenase gradually induced the NGF release.^42^ These results exhibited that the loading various concentrations of NGF in AMHs affected the release patterns.

To investigate the gradient in a conduit in vivo, brain‐derived neurotrophic factor (BDNF) as a model protein was applied to form a BDNF‐G‐AMH@conduit for evaluating the formation of protein gradient in a conduit since BDNF could be stained by fluorescent receptor kit (ab229395, Abcam) (NGF does not have a corresponding fluorescent antibody to label it). In in vivo study, the BDNF‐G‐AMH@conduit was implanted to SD mice in sciatic nerve defects, and then, the conduit was harvested from mice at 1 day and 4 days postimplantation. Figure S4b,c (Supporting Information) displayed the CLSM images and fluorescence intensity of BDNF from the proximal to distal in a conduit, where BDNF represented as red. As the CLSM image shown, the fluorescence intensity of BDNF was weak at the proximal section of the conduit, and the obvious increased intensity of BDNF could be detected from the proximal to distal, indicating the BDNF gradient formation in a conduit at 1 day and 4 days postimplantation.

PC12 cells, derived from a pheochromocytoma of the rat adrenal medulla, were incubated with NGF‐loaded building blocks to evaluate the activity of NGF. The neurite outgrowth of PC12 cells can be induced by NGF through activating the receptor tyrosine kinase, G protein‐coupled receptors and heterotrimeric G proteins. After 2 weeks of incubation, the neurite outgrowth of PC12 cells can be clearly observed on the building blocks, revealing the considerable activity of NGF in AMH (Figure S5, Supporting Information). In addition, the building blocks could also be degraded by human adipose stem cells (hADSCs) in vitro without collagenase after 26 days (Figure S6, Supporting Information).

By directly mixing equal volumes of GelMA and ChitoMA building blocks with identical sizes, the rapid in situ gelation can be accomplished to form a solid hydrogel, which could be further reshaped by an appropriate shear‐thinning force (Figure 2i). This phenomenon implicates that the adaptable interactions can be spontaneously rebuilt through physical crosslinking, known as adaptable hydrogel. To evaluate the void space of AMH, the AMH was immersed to a fluorescence solution (50 kDa of fluorescent dextran) in advance to fill the void volume, and the CLSM images of AMH and unoccupied volume were analyzed to estimate a void fraction distribution (Figure S7a, Supporting Information). As shown in Figure S7b,c (Supporting Information), the various z‐stacks of AMH were analyzed by Zeiss Zen software (blue edition) to determine the total void volume for each layer, and then the results were converted to an average void volume. The result showed an average void volume of approximate 50% for both 125 and 175 µm of building blocks since both building blocks are randomly packed (Figure 2j, left). Furthermore, the interconnected pores with similar patterns can be observed by fluorescence images (Figure 2j, right), suggesting the easy penetration of solution into gel. With an increase of sizes of building blocks from 115 to 210 µm, the void pore diameters could be enlarged from 48 to 80 µm (Figure 2k). Due to the highly porous structure and adaptable gelation, the hydrogels were termed as adaptable microporous hydrogel (AHM), which offered a compact structure and the interconnected pore (Movie S1, Supporting Information).

### 2.2 Rheological and Self‐Healing Properties of AMH

**Figure 3:**
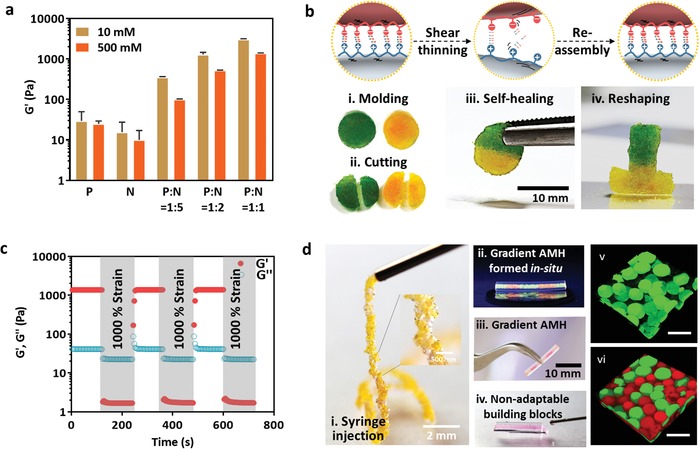
Rheological properties and self‐healing behaviors of AMHs. a) *G*′ of AMH in different volume ratio as a function of the ionic strength. b) Schematic illustration of adaptable process electrophoretic adhesion. Cationic ChitoMA and anionic GelMA are represented in brown and gray, respectively. (i–iv) Images demonstrating self‐healing process and reshapable property of the AMH during electrophoresis. The scale bar is 10 mm. c) Multiple cycles strain oscillatory measurement for self‐healing behavior of AMH. d) (i) Injection of a AMH from a syringe through a 26 G needle. (ii,iii) Five segments of AMH loaded by different dyes were placed into a conduit through a syringe injection. (iv) Nonadaptable gel injected to a conduit. (v, vi) The CLSM images of anionic and cationic building blocks in AMH stained by red and green fluorescent dye. (The scale bar is 200 µm.)

To evaluate the rheological characterization of building blocks and AMH, the bulky storage moduli of building blocks and AMH were investigated through an amplitude sweep (0.01–10% strain) within the linear range. Both storage modulus (*G*′) and loss modulus (*G*′′) were evaluated by an oscillatory time sweep test for 10 min at a stress of 1 Pa with a constant frequency of 1 Hz using a rheometer. In **Figure** **3**a, either positively or negatively charged building blocks displayed a low storage modulus (*G*′ = 20 Pa) since the interactions between building blocks are merely weak van der Waals force in deionic water and PBS solution. Upon mixing two types of building blocks, the storage modulus (*G*′) of AMH was improved to 100 to 3100 Pa, indicating that the strong interactions of the spontaneous adaptability. At various ratios of oppositely charged building blocks (P/N ratio), the wide range of moduli could be observed. For example, the *G*′ of the AMH exhibited 17.5‐ and 155‐fold higher values, i.e., 350 and 3100 Pa, than the negatively charged hydrogels along at P/N = 1/5 and 1/1, respectively. The reason of lower *G*′ of AMH in PBS solution than that in water is dominated by the ionic strength. In PBS solution, the large amounts of ions would weaken the electrostatic forces between two oppositely charged building blocks. However, the adaptable electrostatic forces are strong enough to maintain the compact AMH in ion‐rich environment.

AMH exhibited specific elastic viscosity via the introduction of reversible noncovalent interactions without any assistance from an additional external trigger. As illustrated in Figure 3b, the electrostatic forces between positive and negative building blocks across the interface autonomously self‐healing cracks and rejoin the two halves of the gel. This phenomenon could be observed by directly placing two AMHs together, were stained by green and yellow dyes, respectively (Figure 3bi–iv). The resulting gels exhibited the excellent adaptability, stability, and reshaping ability. This AMH demonstrated the tunable degrees of cohesiveness by controlling the adaptable electrostatic forces, which make it possible to be packed in the complicated voids. By modifying the P/N ratios, AHM could performed distinct rheological properties. For example, AMH possessed an excellent stability in water at P/N = 1/1 (Movie S2, Supporting Information).

The elastic viscosity of AMH (at P/N = 1/1) was validated by the multicycle step strain oscillatory measurements at constant frequency of 1 Hz (Figure 3c). At the first cycle, AMH exhibited an elastic and viscous modulus of 1500 and 42 Pa, respectively, indicating the elastic regime dominated over the viscous regime. At 1% strain of oscillatory shear, elastic modulus (*G*′) is stronger than viscous modulus (*G*″), and they performed constantly at the time duration, suggesting the AMH remained intact at low shear strain. With an rapid increase of shear strain to 1000%, the viscous modulus is much higher than elastic modulus, implying that the viscous regime dominated over elastic at this time period. On the reversal of shear strain, the elastic regime restored its predominant over the viscous domain. The result significantly confirmed the reformation ability of AMH, which also demonstrated the self‐healing nature of AMH. Furthermore, the repeated high strain up to 1000% also displayed no obviously loss in moduli upon the abrupt retraction of elastic domain. AMH regains the original *G*′ and *G*″ within few seconds after deformation because of the rapid adaptable interactions. The repeated oscillatory shear strain of AMH causes low change in moduli, indicating the excellent self‐healing characteristic with a repeatable feature.

AMH were able to be filled in a syringe to pretest its ability for injection through a needle. Figure 3di exhibits that the AMH could successfully be extruded from a syringe through a 26 G needle. At a closer observation, the pearl‐like gel was formed due to the strong interactions between oppositely charged building blocks. Furthermore, five segments of AMH loaded by different dyes were filled in a syringe, and subsequently, injected to a conduit (Figure 3dii). Under an ultraviolet lamp, five parts of AMH with clear boundary was observed. With flipping the conduit, the AMH was still intact without pouring (Figure 3diii). However, once the conduit was filled by the nonadaptable building blocks (GelMA building block only), the poor stability in a conduit would lead to building blocks flow‐out from the conduit owing to the low viscosity as well as the mixing of building blocks in a conduit during the surgery, which was difficult to construct the multiple sections in a conduit for the gradient filling (Figure 3div). The confocal microscopy images of AMH (Figure 3dv,vi) revealed its inter‐connecting structures, where the electrostatic forces between positive (green) and negative (red) building blocks across the interface autonomously self‐heal cracks and rejoin the two halves of the gel. The results demonstrated that AMHs have great potential as injectable tissue constructs.

### 2.3 In Vitro 3D Cellular Network in AMH

**Figure 4:**
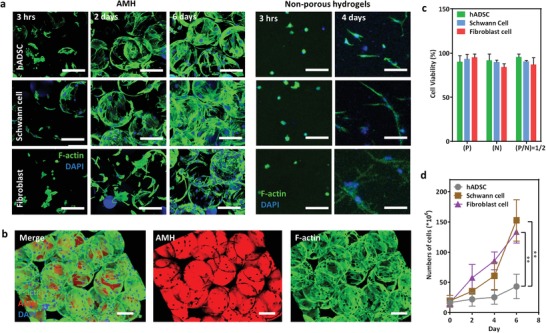
Microporous scaffolds facilitate 3D cellular network formation and proliferation in vitro. a) Proliferation of hADSC, SC and fibroblast cells in AMHs and nonporous gels for various time. The scale bar is 100 µm. b) Fluorescence images demonstrating the formation of 3D cellular networks in AMH in vitro for 6 days. The scale bar is 100 µm. c) Cell viability of survival hADSC, SC, and fibroblast cells in AMHs for 24 h. N and P means the negatively charged and positively charged building blocks, respectively. P/N ratio indicates the weight ratio of positively charged building blocks to negatively charged building blocks. No significant difference for each group (*n* = 5). d) hADSC, SC, and fibroblast cells proliferate within the microporous scaffold for 6 days. Statistical significance performed using one‐way ANOVA with Tukey's multiple comparison test (*n* = 6, *p < 0.05, **p < 0.01).

The ability of AMH to facilitate cell proliferation and network formation was evaluated by incubating three cell types, including SCs, hADSCs, and fibroblasts in the gels. The three cells readily adhered directly on the building block surface within 3 h and proliferated without additional steps for protein adhesion or attachment (**Figure** **4**a), demonstrating the innate cytocompatibility of the AMHs. As the incubation time increased to 2 and 6 days, the SCs and fibroblasts on AMHs demonstrated continued proliferation, and the observed cells in the scaffold displayed a spread and network morphology. However, hADSCs exhibited slower proliferation with an incubation time of more than 6 days. However, cells proliferating in nonporous hydrogels with identical properties (10 wt% GelMA) showed no increase in cell spreading, even after 4 days of culture (right panel of Figure 4a). With increasing the incubation time to 6 days, most of the AMH's surfaces could be covered by hADSCs, indicating the excellent affinity between AMH and cells (Figure 4b). Furthermore, the surface charge effects of AMH on cell viability were also evaluated. Positive building blocks (P), negative building blocks (N), and AMH were incubated with three cell lines for 24 h (Figure 4c). P/N ratio indicates the weight ratio of positively charged building blocks to negatively charged building blocks. The cell viability was more than 90% for the building blocks and AHM for three cell lines, suggesting their low toxicity to cells even through the differences of surface charges of AMH.

The proliferation of SCs in AMH and a 2D cell culture dish was also monitored for 6 days. The proliferation rates of SCs in AMH and cell culture dish were almost the same at the beginning of 4 days (Figure S8a, Supporting Information). However, at sixth day, the numbers of SCs in AMH was ≈1.6‐folds greater than that in culture dish. When compared the proliferation of SCs and fibroblast cells in AMH and nonporous hydrogel, the cell numbers for both cell lines in AMH were higher than that in nonporous hydrogels (Figure S8b, Supporting Information). The improvement of cell proliferation in AMH was potentially attributed by the suitable void volume of AMH for cell adhesion and nutrient transportation. Furthermore, the SC and fibroblast cell lines exhibited continued proliferation over 6 days, with a threefold increase at 2 days (Figure 4d). Cell proliferation in AHM could potentially overcome the limitations associated with 2D cell culture and boost cells for several folds for tissue engineering and regenerative medicine.

### 2.4 In Vivo Study of Regenerated Nerve

**Figure 5:**
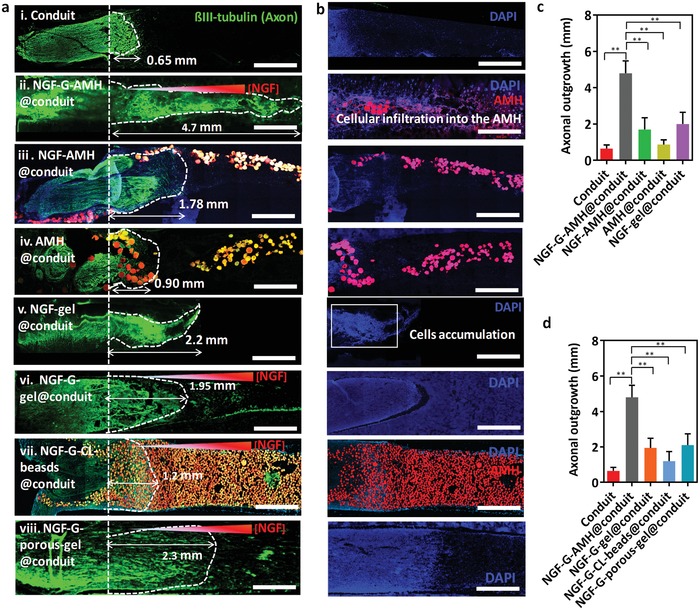
a) In vivo study of immunohistochemistry images of the peripheral nerve regeneration in sciatic nerve defects harvested from (i) conduit, (ii) NGF‐G‐AMH@conduit, (iii) NGF‐AMH@conduit (homogeneous distribution of NGF), (iv) AMH@conduit, (v) NGF‐gel@conduit, (vi) NGF‐G‐gel@conduit (filled by nonporous GelMA gel with NGF gradient), (vii) NGF‐G‐CL‐beads@conduit (filled by crosslinked GelMA building blocks with NGF gradient), and (viii) NGF‐G‐porous‐gel@conduit (filled by porous GelMA gel formed by freeze‐drying). ß‐III tubulin represented as green for regenerated axons and AMH showed in red. The white dashed lines mark the leading edges of the axon outgrowth. (The scale bar represents 1 mm.) b) The immunohistochemistry images of AMH and cells in the conduit for each group, where DAPI displayed as blue for nuclei. The scale bar represents 1 mm. c,d) The axonal outgrowth of each treatment after 4 days. Statistical significance performed using one‐way ANOVA with Tukey's multiple comparison test (*n* = 5 for each treatment, mean ± s.d., **p < 0.01).

To evaluate in vivo study of AMH in a conduit, the conduit with a thickness of 1 mm was prepared in advance by photocrosslinked GelMA through a molding and freeze‐dried process (Figure S10 in Supporting Information). Then, three types of AMHs containing 200, 150, and 100 ng mL^−1^ of NGF‐loaded GelMA and empty ChitoMA building blocks at equal volume were injected into the conduit to propagate the NGF gradient, hereafter referred to NGF‐G‐AMH@conduit, for the implantation to a peripheral nerve regeneration after 5 mm of sciatic nerve transection in SD rats (**Figure** **5**a). Furthermore, other groups including conduit (nonfilled), NGF‐AMH@conduit (filled by homogeneous NGF‐distributed AMH), AMH@conduit, NGF‐gel@conduit (filled by homogeneous NGF‐distributed nonporous gel), NGF‐G‐gel@conduit (filled by nonporous GelMA gel with NGF gradient) and NGF‐G‐CL‐beads@conduit (filled by crosslinked GelMA building blocks with NGF gradient) were also implanted in vivo for a peripheral nerve regeneration, where the total amount of NGF in each group is identical for comparison (the concentration of NGF for homogeneous NGF was 150 ng mL^−1^). At 4 days postimplantation, the harvested conduit with regenerative nerve was fixed overnight in 4% paraformaldehyde in PBS. The conduit with regenerative nerve was sectioned in optimum cutting temperature compound, sliced, and stained for immunohistochemical analysis.

Figure 5a,b exhibits the immunohistochemistry images of sciatic nerve defects in a conduit, where ß‐III tubulin represented as green for regenerated axons, DAPI displayed as blue for nuclei and AMH showed in red. Several conclusions can be drawn from these results. First, the length of regenerated nerve in the conduit alone was the shortest compared to other groups since there was no any support for the SC migration or biocues for guiding (Figure 5ai). Second, ≈4.7 mm of axons was induced by the guidance of NGF‐G‐AMH group within 4 days (Figure 5aii). The positive result could be potentially conducted to the gradient NGF propagation and the interconnected pores of AMH, which could actuate the function of the bands of Büngner for SC migration and guide the direction of axons to shorten the repair time.

To understand the effect on NGF gradient, the nongradient and gradient NGF‐loaded AMHs in the conduits were compared with similar total amounts of NGF in Figure 5aiii. Without the NGF gradient, there was only 1.78 mm of nerve growth in the conduit, which the nerve length is ≈38% compared to the NGF gradient group (i.e., NGF‐G‐AMH@conduit), indicating the guidance effect of gradient NGF propagation. Furthermore, the AMH@conduit group did not obviously improve for nerve regeneration without the assistance of NGF since it is a key biocues for nerve regeneration (Figure 5aiv). Furthermore, in the NGF‐gel@conduit (nonporous gel) group, the cells aggregated at the stump edge near the interface between the nerve defect and bulk gel (Figure 5av), indicating the potential inhibition of the infiltration of cells in the nonporous hydrogel. In Figure 5b, the distribution of cells also exhibited the related cell intrafiltration in the conduit for each group. The cells demonstrated the wide‐ranged distribution through whole conduit in the NGF‐G‐AMH@conduit group. However, in the NGF‐gel@conduit group, most cells were accumulated at the proximal section.

The nerve regeneration by NGF‐G‐gel@conduit and NGF‐G‐CL‐beads@conduit was also evaluated the NGF‐gradient effects in different pore structures. In NGF‐G‐gel@conduit group, about 1.95 mm of nerve growth was observed in the conduit, which the nerve length is about 41% compared to the NGF‐G‐AMH@conduit (Figure 5avi). As the image shown, the cells aggregated at the stump edge of conduit. The results were caused by the low infiltration of cells in the nonporous hydrogel. As literature documented, a mismatch between the material degradation and the rate of tissue development might restrict the cell migration in the tissue regeneration.^43^ In Figure 5avii, the NGF‐G‐CL‐beads@conduit did not exhibit clear improvement in nerve regeneration and the nerve length was ≈1.2 mm after 4 days of implantation. After the crosslinking process, the rate of degradation of GelMA was decreased due to the formation of covalent bonds on the gel surface, which could reduce the release of NGF from gel. To evaluate the crosslinking effects, the NGF‐G‐CL‐beads were incubated with PC12 for 2 weeks. After the incubation, only few PC12 cells were differentiated with neurite (Figure S11 in the Supporting Information). Furthermore, NGF‐G‐porous‐gel@conduit (filled by porous GelMA gel formed by freeze‐drying) was also implanted to SD mice in sciatic nerve defects for a peripheral nerve regeneration (Figure 5aviii). The nerve length for NGF‐G‐porous‐gel@conduit was ≈2.3 mm. The possible mechanism might be caused by the short interconnected pores in the structure.

In Figure 5b, the distribution of cells also exhibited the related cell intrafiltration in the conduit for each group. The cells demonstrated the wide‐ranged distribution through whole conduit in the NGF‐G‐AMH@conduit group. However, in the NGF‐gel@conduit group, most cells were accumulated at the proximal section. The average lengths of nerve regeneration were also provided. Compared to other groups, the NGF‐G‐AMH@conduit with the interconnected porous channels had the longer infiltrated cell distance and exhibited sevenfold longer than that in the conduit group (Figure 5c). In Figure 5d, for the average lengths of nerve regeneration for various NGF‐gradient porous gels in the conduits, NGF‐G‐AMH@conduit with shear thinning/adaptable properties and the interconnected porous channels also showed the longer infiltrated cell distance than that in the other groups.

**Figure 6:**
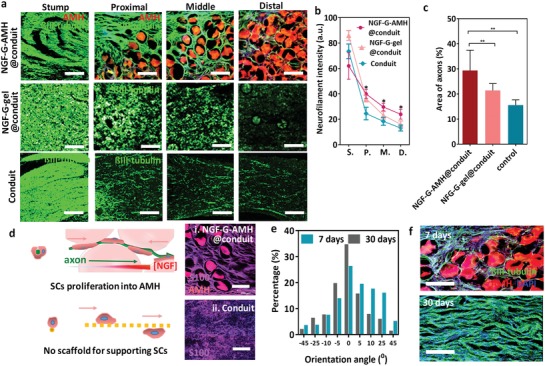
a) CLSM images of axons by staining ßIII‐tubulin in green in the indicated regions of regenerated nerves in NGF‐G‐AMH@conduit, NGF‐G‐gel@conduit, and conduit at 7 days postimplantation. (The scale bar represents 250 µm.) b) Quantification of axonal growth in NGF‐G‐AMH@conduit, NGF‐G‐gel@conduit and conduit at representative segments in the proximal, middle, and distal sections of the nerve gap (*n* = 5, mean ± s.d., *p < 0.05, **p < 0.01, *t‐*test). c) Relative area of axons from proximal to distal (*n* = 5, mean ± s.d., *p < 0.05, *t‐*test). d) Schematic illustration of SCs in AMHs (left). S100 staining displayed the SCs in purple in the (i) NGF‐GAMH@conduit and (ii) conduit. The scale bar represents 250 µm. e) Quantification of the orientation angle of axons from the proximal to distal. Immunohistochemistry images of the NGF‐G‐AMH@conduit at 7 and 30 days postsurgery. f) The axons in green penetrated through the negative space of degradable AMH at day 7. After 30 days, AMH was completely degraded and the axons paralleled in the distal section of a conduit. The scale bar represents 200 µm.

To understand the densities of axons in the conduit, the cross‐sections of regenerated nerves including the stump, proximal, middle and distal parts in the NGF‐G‐AMH@conduit were evaluated by CLSM at 7 days postimplantation. The axons were stained by ß‐III tubulin as green, and AMH was exhibited in red. **Figure** **6**a revealed that the infiltrated cells were appeared in the proximal, middle, and distal sections in NGF‐G‐AMH@conduit because it offered the interconnected porous channels. In NGF‐G‐gel@conduit, the amounts of cells could also be observed in the proximal and middle section of conduit, but the fluorescence intensity from cells was not as strong as that in NGF‐G‐AMH@conduit. However, without the assistance of AMH or gel, the signals of axons became weaker in middle and distal sections. This finding corresponded to the previous in vitro results in Figure 4a, suggesting the cell mobility in the porous network.

The distribution of axons in the NGF‐G‐AMH@conduit for whole regenerated nerve displayed the effects of AMH for nerve growth (Figure S12 in Supporting Information). The nerve filament intensity in NGF‐G‐AMH@conduit exhibited approximately 1.7‐ and 1.4‐ folds greater than that in conduit and NGF‐G‐gel@conduit, respectively, from proximal to distal section, suggesting a robust and steady rate of axon outgrow in NGF‐G‐AMH@conduit (Figure 6b). Consistent to the densities of axons, the area of axons in NGF‐G‐AMH@conduit was also larger than that in conduit and NGF‐G‐gel@conduit (Figure 6c). Furthermore, at 4 days postimplantation, both conduit and AHM@conduit were stained by CD68, a maker for macrophage, to track the immune response in the area of proximal stump and nerve gap. As shown in Figure S13 in Supporting Information, the lower fluorescence signal of CD68 was present in the AMH@conduit than that in the nerve gap (conduit), indicating that the positive charged building blocks would not cause serious inflammatory. The result is consistent with previous studies displaying the tolerable immune response of gelatin‐ and chitosan‐based materials in vivo.^1^, ^43^, ^44^

SCs play an important role in creating a path as bands of Büngner for promoting axon penetration (Figure 6d). Before the nerve regeneration, the SCs have to migrate into the subtract and form a fibrin‐like structures to guide the axons. Therefore, the proliferation of SCs in the NGF‐G‐AMH@conduit was also investigated. At 7 days postimplantation, the fibrin‐like morphology of SCs with high density was observed in NGF‐G‐AMH@conduit by confocal microscope in Figure 6d, where the SCs were stained by S100 in purple, indicating that the quick infiltration of SCs into the gradient AMH. However, the fibrin structure of SCs in conduit alone was not clear. Through such effective guidance of SCs, the orientation of axons at the distal sections revealed that most of axon morphology followed the direction of conduits (i.e., the orientation angle was 0°) in Figure 6e. At seventh day, the degradation of AMH was occurred with the infiltration and regeneration of cells (Figure 6f). After one month, the NGF‐G‐AMH significantly was degraded in vivo, and axons preformed into a regular arrangement and uniform size, illustrating that the AMH had the ability to construct the bridge structure necessary for the peripheral nerve generation (Figure 6f).

The degradation of AMH was dominated by collagenase preparations, mammalian MMP‐2, and MMP‐9 through the enzymatic degradability of gelatin preserved from the polymerization and modification with methacrylate pendant groups.^42^ The finding to the rapid adaptation of interconnected porous and gradient scaffold allows SC migration to AMH without compromising the nonporous integrity of hydrogel and promotes axons outgrow directly.

### 2.5 Functional Recovery of a Regenerated Sciatic Nerve In Vivo

**Figure 7:**
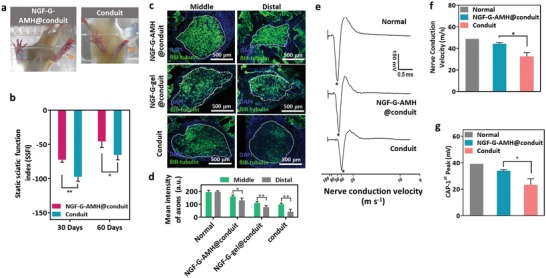
Functional recovery of a regenerated sciatic nerve in vivo. a) Plantar views of the hind paws of animals receiving the implantation of the conduit and the NGF‐GAMH@conduit after 1 month. (The yellow arrowheads indicate the injured hind limbs.) b) Static sciatic function index (SSFI) after implantation for 1 month and 2 months (*n* = 5, mean ± s.d., *p < 0.05, **p < 0.01, *t‐*test). c) ßIII‐tubulin (green) staining represents the regenerative axons. The segments in the middle and distal sections of the nerve gap were collected for analysis. d) The mean intensity of axonal growth in the distal section represents the numbers of axon fibers of the regenerative nerve. Statistical significance performed using one‐way ANOVA with Tukey's multiple comparison test (*n* = 6, *p < 0.05, **p < 0.01). e–g) Electrophysiological assessments of the regenerated nerves in different treatment groups after 2 months of implantation. Nerve conduction velocity (NCV) and CAP‐1st values were recorded at different intervals after surgery. The normal group refers to data collected from the normal limb (*n* = 5, mean ± s.d., *p < 0.05, **p < 0.01, one‐way ANOVA with Tukey's multiple comparison test).

To evaluate in vivo‐promoted axonal regeneration of a transected sciatic nerve and functional recovery, the toe spreading of an injured hind paw and the static sciatic functional index (SSFI, a typical walking footprint analysis of toe spreading^45^) were measured at 30 days postimplantation. The wider toe spreading of injured hind paw (yellow arrowhead in **Figure** **7**a) was observed in the NGF‐G‐AMH@conduit group than that in the conduit group, indicating the better nerve regeneration with the assistance of NGF‐G‐AMH. By analyzing the SSFI (0 and −100 for the healthy and transected sciatic nerves animals), the average value of the NGF‐G‐AMH@conduit was higher than that of the conduit group at 30 days postsurgery (Figure 7b). The observations revealed that the function‐promoting effect of NGF‐G‐AMH@conduit was better than that in conduit in the nerve regeneration. Furthermore, the nerve fiber density in the NGF‐G‐AMH@conduit was also higher than that in the conduit and NGF‐G‐gel@conduit (Figure 7c). The quantitative mean intensity of axons in the distal and middle parts were 3.1‐ and 1.7‐fold higher for the NGF‐G‐AMH@conduit groups than for the conduit group (Figure 7d).

The gastrocnemius muscle wet weight is another critical factor to evaluate the peripheral nerve regeneration after sciatic nerve transection. Once sciatic nerve injury occurred without treatment, the denervation would cause the atrophy of the gastrocnemius muscle. The relative gastrocnemius muscle wet weights of the injured limbs to the contralateral limbs after one month and two months of the implantation of autograft, NGF‐G‐AMH@conduit and conduit were investigated (Figure S14a, Supporting Information). The gastrocnemius muscles recovered in the NGF‐G‐AM@conduit group after 30 days were close to that in the autograft (gold standards in today's methods of nerve gap repair), indicating the positive effects of NGF gradient and AMH guidance. The fast nerve regeneration of autograft was directly actuated by the nerve bundle and supported tissue, but the limitation was still induced by the critical length and a donor nerve was also required.^46^ The weights of relative gastrocnemius muscle after implanting NGF‐G‐AMH@conduit at various times also exhibited the gradual recovery of nerve and gastrocnemius muscle (Figure S14b, Supporting Information).

To confirm the functional recovery of regenerated sciatic nerves, the nerve conduction velocity (NCV) and the compound action potentials (CAP^1st^, an algebraic sum of all individual fiber action potentials of the nerve) was monitored in Figure 7e. The CAP value is the algebraic sum of all individual fiber action potentials of the nerve and is highly influenced by the axon diameters and thickness of myelin around the nerve fibers. Furthermore, the value of NCV is based on the first peak of CAP and correlated to the mature of regenerative nerve fiber. When compared to normal group (uninjured control), the electronic signal recovery based on NCV was about 89% for the NGF‐G‐AMH@conduit and 67% for conduit alone, respectively (Figure 7f). Similarly, a significantly higher CAP^1st^ signal in the NGF‐G‐AMH@conduit group was detected than that in conduit alone (Figure 7h). These results demonstrated that the NGF‐G‐AMH@conduit not only enhanced the effective restoration of nerve conductivity but also offered the additional functional improvement through NGF‐loaded AHM.

### 2.6 Regenerated Myelinated Nerve Fibers

**Figure 8:**
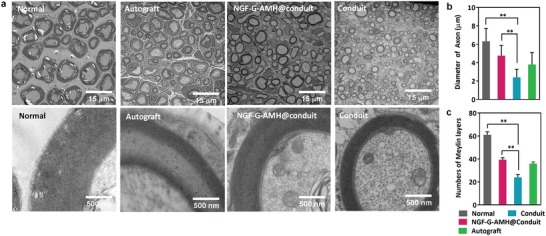
Transmission electron microscopy (TEM) analysis of regenerated distal nerves. a) TEM images of cross sections of regenerated distal nerves taken from types of nerve conduits implanted in rats after 60 days. b) Quantification of average axon diameter of regenerated myelinated nerve fibers. c) Average layers of regenerated myelinated sheath (*n* = 5, mean ± s.d., **p < 0.01, one‐way ANOVA with Tukey's multiple comparison test).

The myelination process of SCs plays an important role in axonal regeneration after PNI since the developing nervous system was surrounded by a myelin sheath. Distal SCs undergo atrophy owing lose axonal contact for a long duration leads to the inhibition of nerve regeneration. Typically, a higher number of distal axons and well myelination, the targeting tissue/organ would seem to perform the best functional recovery for the injured nerve. Transmission electron microscopy (TEM) of the cross section distal regenerative nerve segment at 60 days postimplantation revealed the well‐laminated myelin sheathes of regenerated nerve fibers in the normal and NGF‐G‐AMH@conduit group, whereas those in the conduit group had only small and thin myelin sheathes (**Figure** **8**a). Furthermore, the myelin sheathes of regenerated nerve fibers in NGF‐G‐AMH@conduit was very close to that of the autologous nerve graft group (gold standard) after 2 months of repair. To compare the treatments, the layers of regenerated myelinated fibers were clearly calculated in the cross‐sections of the regenerated nerve fibers. Mean axon diameter and myelin layers were significantly greater in the NGF‐G‐AMH@conduit group (4.76 ± 1.11 µm and 39.4 ± 1.69 layers, respectively) than in the conduit group (2.41 ± 0.87 µm and 24.0 ± 2.42 layers, respectively) as shown in Figure 8b,c. Both mean axon diameter and myelin layers in the NGF‐G‐AMH@conduit group were also close to the autologous nerve graft group (gold standard). The finding indicated that the NGF‐G‐AMH@conduit could enhance not only the diameter of regenerative axon but also the numbers of layers of myelin sheath through the gradient and interconnected pores, which amplified the nerve function recovery and the cell‐infiltrated ability in early nerve‐repairing stage.

## 3 Conclusions

In summary, a versatile adaptable hydrogel allowing incorporation of living cells was developed to serve as an injectable matrix with interconnected pores, tunable stiffness, and controllable pore sizes for spontaneous assembly into a complex shape in real time. These results were made possible by the use of microsized building blocks with opposite charges through microfluidic fabrication. Adaptable hydrogels with suitable micropores provide mechanical support for rapid cell migration and transport of bioresponsive cues to direct cell adhesion and growth. Furthermore, after loading gradient concentrations of growth factors into the building blocks, the propagated gradient of the nerve growth factor, combined with the cell‐penetrative connected pores constructed by the building blocks in the nerve conduit, effectively promotes SC migration and induces dramatic bridging effects on peripheral nerve defects, achieving axon outgrowth of up to 4.7 mm and twice axon fiber intensity within 1 week in vivo. Such adaptable microporous hydrogels with intrinsic properties of tunable mechanical properties, a high drug payload, effective induction of cell migration and biocompatibility are potentially able to overcome the limitations of hydrogel‐mediated tissue regeneration in general and can possibly be used in clinical applications.

## 4 Experimental Section

*Microfluidic Chip Fabrication*: The network of chips was designed by using geometric modeling software (AutoCAD, Autodesk Inc., Sausalito, CA, USA). Designed patterns were generated on the surface of PMMA substrates (Kun Quan Engineering Plastics Co. Ltd., Taiwan) by using a CO_2_ laser micromachining system (LES‐10, Laser Life Co. Ltd., Taiwan). Figure S1 (Supporting Information) shows the layout of the microchip (6.5 cm (*L*) × 3.5 cm (*W*) × 0.4 cm (*H*)), which consisted of two separated PMMA plates. The channel features were observed by using an optical microscope (FS‐880ZU, Ching Hsing Computer‐Tech Ltd., Taiwan). Several access holes were drilled in the PMMA substrates. Each plate was immersed in D.I. water with ultrasonic agitation for 30 min and then dried with a stream of N_2(g)_. The cleaned plates were compressed together and bonded at 105 °C for 0.5 h. Finally, poly(etheretherketone) (PEEK) tubes (Upchurch Scientific Inc., Oak Harbor, WA, USA) were inserted into the holes and fixed by using an AB glue.^47^

*Fabrication of Building Blocks By a Microfluidic Chip*: The aqueous phase (GelMA/ChitoMA solution with 0.5 wt% Irgacure 2959 in D.I. water) and oil phase (5 wt% Span80 in paraffin oil) were injected, and the flow rates were well controlled by syringe pumps. The experimental setup was placed under an inverted optical microscope to monitor the process of droplet formation. The droplets, which flowed through the outlet and the connected glass tubing, were crosslinked to building blocks by UV light (365 nm, Series 1500, OmniCure). The size of the building blocks was determined using microscope images and Nikon software. These building blocks were washed with hexane three times and then centrifuged in PBS to remove the oil and surfactant. After removing the upper organic phase, the building blocks were washed with PBS three times and preserved at 4 °C until further use. Because of the gelling temperature of the prepolymer, these experiments should be performed at room temperature (not below 20 °C). For the fluorescence imaging, the building blocks were dyed red by Rhodamine B isothiocyanate (RITC). Briefly, after the building blocks were polymerized on a microfluidic water‐in‐oil (w/o) chip, they were collected and washed by the excess of deionic water for several time. Then, the building block was redispersed in PBS solution, 0.01% RITC was added in the solution at 4 °C for 3 days. After 3 days, these building blocks were washed with PBS for three times, and then, centrifuged to remove the unreacted RITC and preserved at 4 °C until further use.

*Elastic Modulus Measurement*: The cylindrical GelMA/ChitoMA hydrogels at different concentrations were prepared as previously described.^48^, ^49^ Then, the hydrogels were compressed at a rate of 20% strain min^−1^ on an Instron 5542 mechanical tester. The elastic modulus was determined as the slope of the linear region corresponding to 0−5% strain of a stress−strain curve.

*Degradation of The GelMa Building Blocks*: Lyophilized GelMA building blocks were weighted in 2.0 mL Eppendorf and then swelling in 2.0 mL of 0.5 U collagenase type II solution at 37 °C. Collagenase solution was replaced 1.0 mL every day to ensure constant enzymatic activity. At each time point, the solution was removed from Eppendorf and the building blocks were washed three times with PBS, lyophilized and weighed. The equation, *D*% = (*w*_0_ − *w*_t_)/*w*_0_ × 100%, was used to calculate percentage degradation (*D*), where *w*_0_ was the initial lyophilized GelMA building blocks dry weight and *w*_t_ was the dry weight after time *t*.

*In Vitro NGF Release Study*: To prepare NGF‐loaded GelMA building blocks with 200, 150, and 100 ng mL^−1^ NGF, 2, 1.5, and 1 µL of NGF solution (100 µg mL^−1^) was added to a premade solution containing 10.0 wt% GelMA and 0.5 wt% Irgacure 2959, respectively. After washing, the crosslinked building blocks were 175 µm in diameter in 0.5 U collagenase solution. The loading efficiency and the amount of NGF released from the GelMA microspheres were determined using an NGF rat ELISA Kit (Arigo Biolaboratories Corp). The release profile of NGF from the GelMA building blocks in vitro was investigated for 8 days until the MPs were all degraded. GelMA building blocks (1.0 mL) were pipetted into Eppendorf tubes containing 1.5 mL of 0.5 U collagenase solution at 37 °C. At each time point, 500 µL of storage solution was collected for measurement, and 500 µL of fresh 0.5 U collagenase solution was added to the remaining solution.

*Rheology Technique for The Gel Measurement*: All measurements were performed using a flat steel plate geometry (20 mm diameter) at 25 °C with a gap distance of 0.5 mm using a rheometer (AR 2000ex, TA Instruments). To determine the bulk storage modulus of unmixed building blocks and mixed AMH scaffolds, an amplitude sweep was performed (0.01−10% strain) to find the linear amplitude range for each. An amplitude within the linear range was chosen to run a frequency sweep (0.5–5 Hz). For AMH scaffold measurements, first, each sample of oscillatory stress and strain sweeps was performed on a linear viscoelastic region. Subsequently, the storage modulus *G*′ and loss modulus *G″* were determined using an oscillatory time sweep test for 10 min at a constant stress of 1 Pa and constant frequency of 1 Hz.

*Cell Lines and Animals*: Fibroblast cells (HIG‐82) were obtained from ATCC. Schwann cells (SCs) were isolated from SD rat (National Laboratory animal center, Taiwan). Human adipose stem cells (hADSCs) were kindly provided by Dr. Hui‐Yi Hsiao (Chang Gung Memorial Hospital, Center for Tissue Engineering, Taiwan). HIG‐82 cells were cultured in F‐12 medium containing 10% fetal bovine serum and 100 U mL^−1^ penicillin–streptomycin. The SCs were cultured in DMEM medium (Gibco) containing 0.094 g L^−1^ d‐valine (Sigma‐Aldrich), 10% (v/v) FBS, 1% (v/v) N_2_ supplement (Gibco), 20 µg mL^−1^ (wt/v) bovine pituitary extract (Sigma‐Aldrich), 5 × 10^−6^ m forskolin (Sigma‐Aldrich), and 1% (v/v) penicillin/streptomycin (Gibco). The hADSCs were cultured in DMEM/F‐12 medium (Gibco) containing 10% fetal bovine serum and 100 U mL^−1^ penicillin–streptomycin. All cells were maintained in a 37 °C incubator (Water‐Jacketed 3010 CO_2_) under a humidified atmosphere with a 5% CO_2_ supply. All experiments were performed in the logarithmic phase of cell growth. All animals (SD rats) were purchased from National Laboratory Animal Center, Taiwan.

*Cell Culture on Building Blocks*: The building blocks were incubated in media with serum before being dried and pelleted by centrifugation and aspiration of supernatant. One hundred microliters of building block was added to a 100 µL stock solution of 1.5 × 10^4^ cells mL^−1^ at 37 °C for 90 min in situ for adherence rather than being transferred to the culture dish. Afterward, the cells were stained with DAPI for the nucleus and F‐actin for the cytoskeleton, washed twice with PBS after each staining, and imaged using a laser scanning confocal microscope (ZEISS LSM‐780).

*Cell Proliferation Analysis*: Proliferation was assessed by counting the number of cell nuclei present in the AMH scaffold or nonporous gel constructs after 90 min and 2 and 6 days of culture in vitro. Nuclei were stained with a 2 µg mL^−1^ DAPI solution in PBS for 2 h, followed by visualization on a laser scanning confocal microscope (ZEISS LSM‐780). Specifically, each scaffold was imaged by taking 30 *z* slices over a total *z* height of 250 µm. At each time point, the total number of cells for 10 *z*‐stack images was counted. These data were presented for all three cell lines. The cell viability was evaluated by 100 µL of cells containing 5000 cells per well coincubated with the building blocks on 96‐well plates for 24 h. Then, the cell viability was evaluated by MTS assay and determined by normalizing the results of treated to untreated with building blocks.

*Preparation of GelMA Nerve‐Guiding Conduit*: Prepolymer solution was formed by dissolving 30% (w/v) GelMA with 0.5 wt% Irgacure 2959 in D.I. water at 60 °C. The solution was injected into a conduit mold cavity in a 60 °C oven, which avoided gelation and bubble formation. After 5 min, the molds were removed and quickly cross‐linked by UV light. The resulting cross‐linked NGCs were hydrated by lyophilization and then stored at −20 °C until further use. Before surgery, the NGCs were immersed in dH_2_O for 1 h and autoclaved for sterilization. The swelling ratio, diameter, and SEM morphology are shown in Figure S10 in Supporting Information.

*Morphology of Regenerated Distal Nerves*: The morphology at the surface of the GelMA NGCs was observed by using scanning electron microscopy (SEM, Hitachi S4800) with an accelerating voltage of 5 kV. Prior to observation, the samples were sputter‐coated with gold for 90 s before SEM imaging.

*Transmission Electron Microscopy of the Regenerated Nerves*: The regenerative nerve was fixed at 4 °C with 3% glutaraldehyde, washed in 0.1 m PBS, postfixed with 1% osmium tetroxide (Fisher Scientific, Pittsburgh, PA), dehydrated in graded ethanol solutions, and embedded in Araldite 502 (Polysciences Inc.). Ultrathin sections (60 nm) were lifted onto formvar‐coated grids, poststained with lead citrate and uranyl acetate, and subsequently imaged using electron microscopy.

*Relative Gastrocnemius Muscle Weight (RGMW)*: After performing the experiments, the animals were sacrificed and regenerating nerve was removed for further analysis, the gastrocnemius muscle of both hind limbs was excised and cleaned with PBS. Both right and left gastrocnemius muscle were weighted in order to determine relative gastrocnemius muscle weight.

*Tissue Section Immunofluorescence at 4 And 7 Days Postinjection*: At 4 and 7 days postsurgery, the harvested conduit with regenerative nerve was fixed overnight in 4% paraformaldehyde in 0.1 m phosphate buffered saline (PBS) at pH 7.4. The conduit with regenerative nerve was sectioned and embedded in optimum cutting temperature compound (OCT; Surgipath FSC22, USA). Horizontal cryostat sections (10 µm in thickness) were sliced and then stained for immunohistochemical analysis. In brief, the frozen sections were incubated at room temperature to melt frozen section compound for 30 min. Then Immersed slide in the ethanol at 4 °C to remove frozen section compound for 10 min. Then, the sections were stained overnight at 4 °C with the primary antibody solutions. The obtained slides were stained as follows: 1) ß‐III tubulin (1:200, rabbit IgG1, Abcam) for regenerated axons. 2) S100 (1:200, rabbit polyclonal, Abcam) for Schwann cells. The secondary antibody was stained for 2 h by using Alexa 488 (goat anti‐rabbit IgG1) and then washed three times with PBS. Next, stained the section with DAPI for 10 min and then washed three times with PBS. The morphology of all the stained sections was observed using a laser scanning confocal microscope (ZEISS LSM‐780). The intensity and the alignment of regenerating axons at each segment (proximal, middle and distal) were analyzed by image J software.

*Statistical Analysis*: Statistical analysis was performed using GraphPad Prism software (version 5.0). Unpaired Student's t‐test (two‐tailed) was used to compare mean values of two groups. One‐way ANOVA followed by Tukey's post hoc analysis was used for the mean comparison of three groups and more. Values are expressed as means ± standard deviation. A value of *p* < 0.05 was considered statistically significant.

## Conflict of Interest

The authors declare no conflict of interest.

## Supporting information

Supplementary

Supplementary

Supplementary
