# Supplementary material for: Adaptable Microporous Hydrogels of Propagating NGF‐Gradient by Injectable Building Blocks for Accelerated Axonal Outgrowth
Source: Adv Sci (Weinh). 2019 Jul 11;6(16):1900520. doi: 10.1002/advs.201900520 (PMC6702647; doi:10.1002/advs.201900520)
Supplement: Supplementary file 1 — Supplementary [file ADVS-6-1900520-s001.pdf]

Copyright WILEY-VCH Verlag GmbH & Co. KGaA, 69469 Weinheim, Germany, 2019.

Supporting Information

for *Adv. Sci.,* DOI: 10.1002/advs.201900520

Adaptable Microporous Hydrogels of Propagating NGF-Gradient by Injectable Building Blocks for Accelerated Axonal Outgrowth

Ru-Siou Hsu, Pei-Yueh Chen, Jen-Hung Fang, You-Yin Chen, Chien-Wen Chang, Yu-Jen Lu, and Shang-Hsiu Hu**

Supporting Information

Adaptable Microporous Hydrogels of Propagating NGF-Gradient by Injectable Building Blocks for Accelerated Axonal Outgrowth

Ru-Siou Hsu[1], Pei-Yueh Chen[1], Jen-Hung Fang[1], You-Yin Chen[2], Chien-Wen Chang[1], Yu-Jen Lu[3,*], Shang-Hsiu Hu[1,*]

[1]Department of Biomedical Engineering and Environmental Sciences, National Tsing Hua University, Hsinchu, Taiwan.
[2]Department of Biomedical Engineering, National Yang Ming University, Taipei, Taiwan.
[3]Department of Neurosurgery, Chang Gung Memorial Hospital, Linkou, Taiwan.

Experimental Section

Synthesis of GelMA. Briefly, 5.0 g of type B bovine skin gelatin (Sigma-Aldrich) (10 % wt/v) was dissolved in 50 mL of deionized water and mixed by a magnetic stirrer at 60 °C. Then, 1.0, 2.5 or 5.0 mL of methacrylic anhydride was slowly added to the gelatin solution over 3 h at 60 °C. The reaction was stopped by diluting the solution to a 5-fold volume. The solution was dialyzed in tubing with a 12–14 kDa cutoff at 40 °C twice a day for 5 days to remove unreacted methacrylic anhydride (MA) and salts. The solution was lyophilized for 4 days until a white porous foam was obtained, and the foam was stored at −20 °C. By using ^{1}H NMR (Bruker 500) at 40°C, integrated areas the peak at 7.4 ppm, the peak at 3.1 ppm and the peaks at 5.5 and 5.7 ppm combined, which represent the number of aromatic residues of gelatin, the number of amine groups in gelatin and the number of methacrylamide groups, respectively, were obtained. The methacryloyl substitution of the gelatin was confirmed by ^{1}H NMR analysis as previously described[S1] (Figure S2, Supporting Information).

Synthesis of ChitoMA. Similarly, chitosan methacrylate (ChitoMA) was synthesized as previously described[S2]. Initially, 3.0 g of deacetylated chitosan (Sigma-Aldrich) (3% wt/v) was dissolved in 100 mL of 1 M acetic acid solution overnight, and 9.0 mL of MA was added to the chitosan solution for 5 h at 50°C. The reaction was stopped by diluting the solution to a 5-fold volume. The solution was dialyzed in tubing with a 12–14 kDa cutoff at 40 °C twice a day for 5 days to remove unreacted methacrylic anhydride and salts. The solution was lyophilized for 4 days until a white porous foam was obtained, and the foam was stored at −20 °C. The DM of ChitoMA was calculated from the ratio between the integrated area of the signals of H_2–H_6 protons of chitosan glucosamine and N-acetyl glucosamine residues and that of methylene peaks (Figure S2, Supporting Information).

Zeta Potential Measurement. The zeta potentials of GelMA and ChitoMA were measured using laser Doppler electrophoresis with dynamic light scattering (DLS) on a Nanosizer instrument (Zetasizer Nano ZS) by dispersing prepolymer in buffer at different pH values.

Measurement of the Porosity of AMH. To determine the porosity of the AMH, stocks of different sized building blocks in 10^{-6} M FITC-glucose (MW: 500 kDa) solution were used. Using a laser scanning confocal microscope (ZEISS LSM-780), 10 z-slices were taken in each gel, spanning a total distance of 250 μm. The images were analyzed to determine the individual pore areas and total 2D void space using a custom Matlab program. To obtain the characteristic pore length, we treated the pore areas mathematically as circles, and the diameters of these circles were calculated.

Measurement of the Median Void Volume Size in AMH. To determine the median pore size in the AMH, stock solutions of different sized building blocks were used. Using a laser scanning confocal microscope (ZEISS LSM-780), 8 z-slices were taken of each gel, spanning a total depth of 150 μm. These images were then analyzed using ZEISS LSM-780 software to identify the pore regions and calculate each pore size in px^2. Each individual pore's size was then used to calculate the median pore size for that gel and converted to μm^2 using the pixel-to-μm conversion from the original microscope image. These areas were then converted to a characteristic length measurement by forcing the areas to a circle and calculating the characteristic diameter of these circles.

Animal and Surgical Procedure. All surgical procedures were performed in accordance with the protocol approved by the Animal Care and Use Committee, National Tsing Hua University, Hsinchu, Taiwan. The GelMA conduits were immersed in PBS for 2 h for reaching fully swelling. Sprague-Dawley (SD) (male, 220–250 g) were divided into eight groups (n=5 for each group) for the peripheral nerve regeneration study in sciatic nerve defects. Eight types of conduits including (i) conduit (non-filled), (ii) NGF-G-AMH@conduit (three types of AMHs containing 200, 150, and 100 ng mL-1 of NGF-loaded GelMA and empty ChitoMA building blocks at equal volume were injected into the conduit to propagate the NGF gradient), (iii) NGF-AMH@conduit (filled by homogeneous NGF-distributed AMH), (iv) AMH@conduit, (v) NGF-gel@conduit (filled by homogeneous NGF-distributed non-porous gel), (vi) NGF-G-gel@conduit (filled by non-porous GelMA gel with NGF gradient), (vii) NGF-G-CL-beads@conduit (filled by cresslinked GelMA building blocks with NGF gradient), and (viii) NGF-G-porous-gel@conduit (filled by porous GelMA gel formed by freeze-drying) were implanted *in vivo* for a peripheral nerve regeneration (n=5 for each group). To prepare the (vi) NGF-G-gel@conduit, three parts of GelMA containing NGF with the concentrations of 200, 150, and 100 ng mL^{-1} were cured in a conduit subsequently. Furthermore, (vii) NGF-G-CL-

beads@conduit was fabricated by loading AMHs containing 200, 150, and 100 ng mL^{-1} of NGF-loaded GelMA and empty ChitoMA building blocks to the conduit, and then, cross-linked by 1-ethyl-3-(3-dimethylaminopropyl)carbodiimide hydrochloride (EDC) in phosphate buffers (pH=7.2). After the crosslinking reaction, the gel could be fixed in the conduit and the excess crosslinking agent was removed by de-ionic water.

All the rats were anesthetized with 4% isoflurane using a nasal mask) connected to a Univentor 400 Anesthesia Unit. 5-mm defect in the sciatic nerve on the left hind limb created by surgery. In animal surgery, both the proximal and distal stumps of a sciatic nerve were sutured using 9–0 nylon surgical sutures to a depth of 1mm into the GelMA conduits.

Functional Assessment of Walking Track Analysis. The animals were assessed using the footprint analysis each week after the surgery. The feet of the injured limb and the contralateral were photographed and the images were analyzed using the software Image-Pro Plus (version 6.0.0.260, Media Cybernetics, Inc., MD, USA) with these parameters: (1) TS (toe spread), the distance between the first and the fifth toes; (2) ITS (intermediate toe spread), the distance between the second and the fourth toes. (3) PL (print length) is the distance from the heel to the third toe. Static sciatic functional index (SSFI) was calculated as previously described[S3] by the formula proposed by Bain et al. as follows: SSFI = −38.3(EPL − NPL)/NPL + 109.5(ETS − NTS)/NTS + 13.3(EIT − NIT)/NIT − 8.8. (N: the contralateral hind limb; E: the experimental hind limb; A value of −100 implies total impairment.)

Electrophysiological Analysis. Electrophysiological tests were performed using a previous developed method at 2 months after implantation by electromyography machine (Nuocheng, Shanghai, China). The recording electrodes were placed as described above. Using a bipolar steel hook electrode, the sciatic nerves were directly stimulated proximal and distal to the transplants. Single electrical pulses (100 ms duration, not exceeding 4 mA) were applied in order to evaluate the $CAP1^{st}$ and the NCV value (with a current intensity of 1 mA).

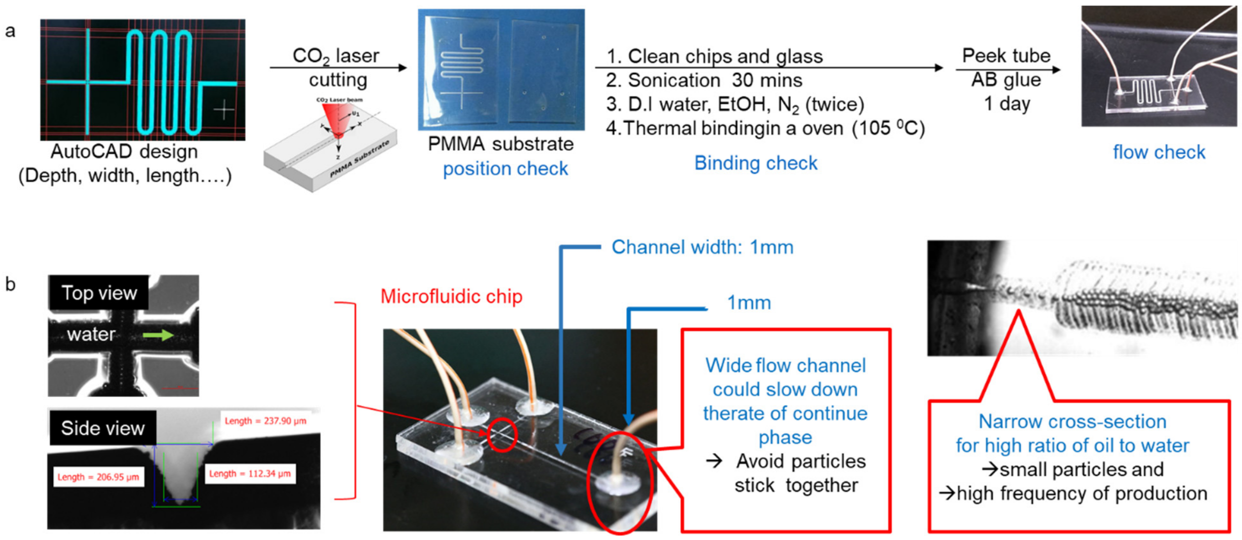

Figure S1. The process of microfluidic chip fabrication. a) Scheme of the microfluidic channel design used, with two aqueous inlets and two oil inlets. b) Images of top view and side view of the cross-section channel. In the droplet segmentation region, mineral oil with 5% Span 80 pinches and segments pre-gel in paraffin oil.

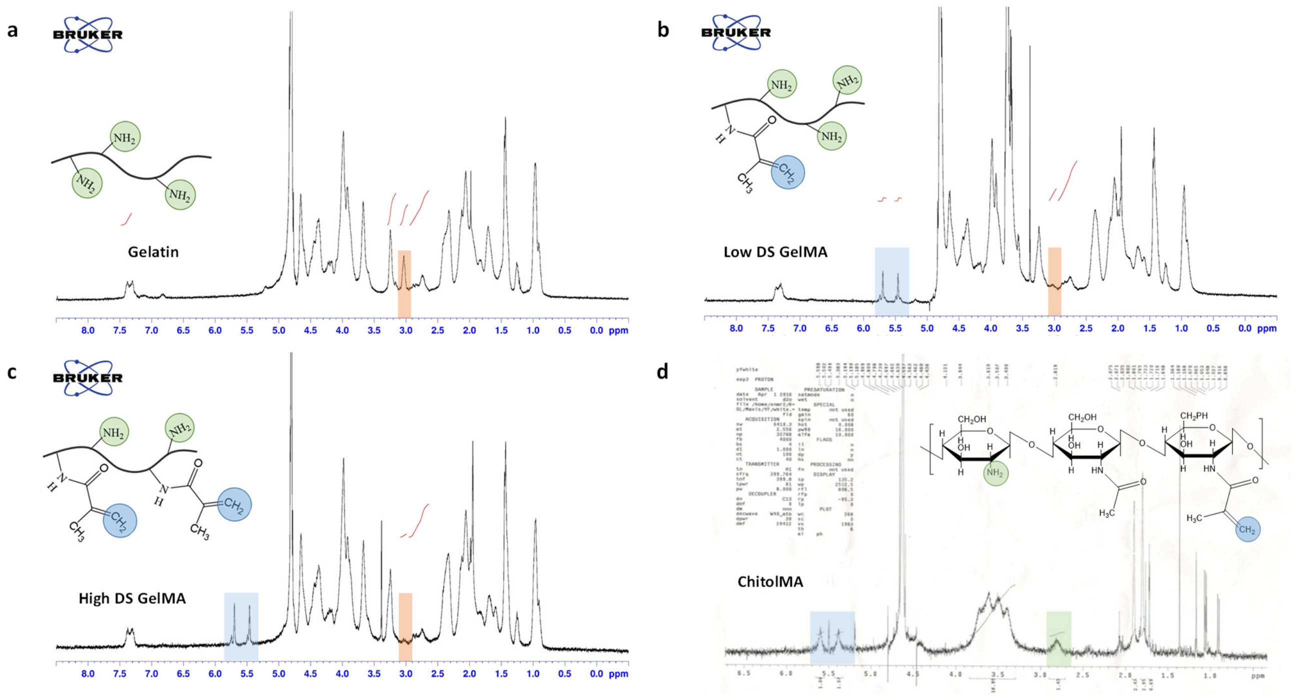

e

	GelMA(1:1)	GelMA(5:1)	ChitoMA
Degree of substitution (%)	84	51	30

Figure S2. H^1-NMR spectrum of GelMA. Peaks corresponding to methacrylate are noted (a to c). a)

Un-modified gelatin, b) Gelatin:methacrylate = 5:1, c) Gelatin:methacrylate = 1:1, and d) Chitosan:methacrylate = 1:3. e The degree of substitution with different ratio reactants.

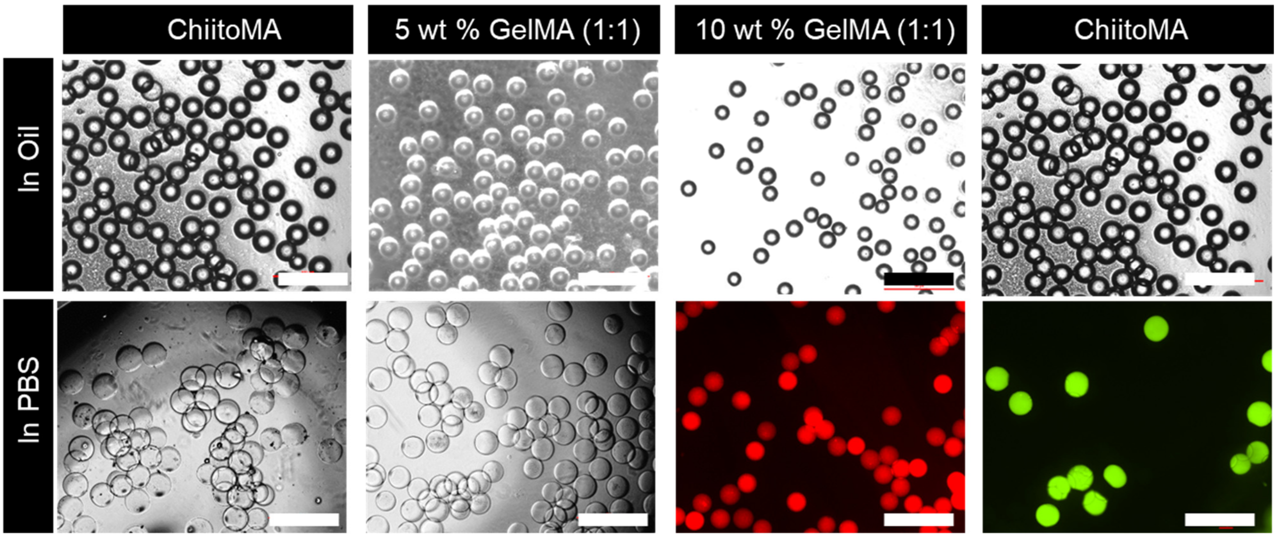

Figure S3. Building blocks were re-dispersed and swelled in PBS buffer after aqueous extraction from the oil phase. Scale bar is represented as 500 μm.

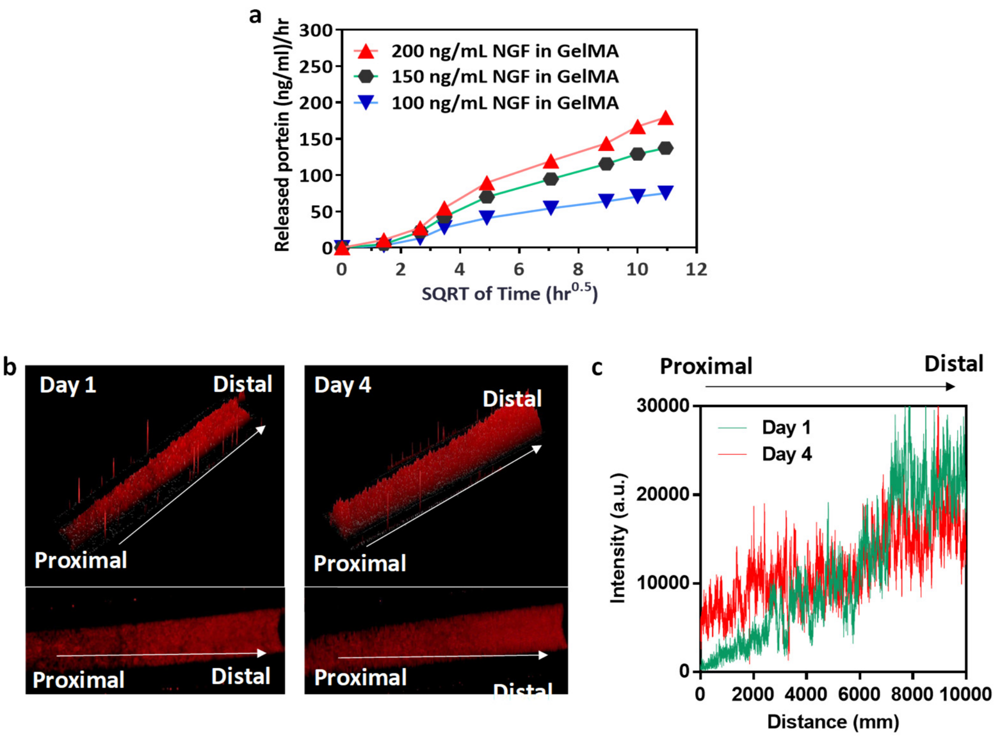

Figure S4. a) The cumulative protein release with square root of time from 10 wt% GelMA. b,c) The CLSM images and fluorescence intensity of BDNF-G-AMH in the conduits at 1 day and 4 days post-implantation, where the conduits were implanted to SD mice in sciatic nerve defects, and then, the conduit was harvested from mice at 1 day and 4 days post-implantation.

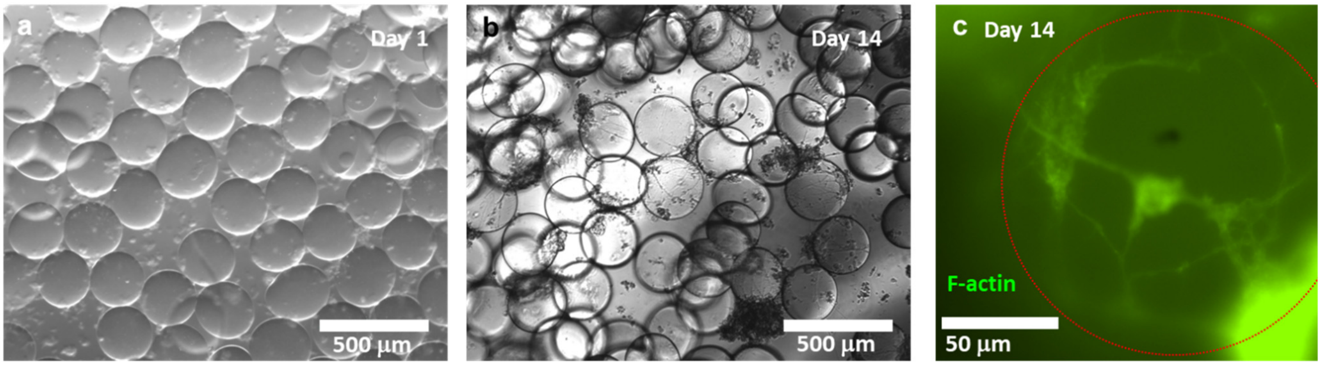

Figure S5. The stability of NGF inside the 10 wt% GelMA building blocks with PC12 Cells. Released NGF from building blocks stimulated the axon outgrowth of PC12 after a) 1 day and b,c) 14 days.

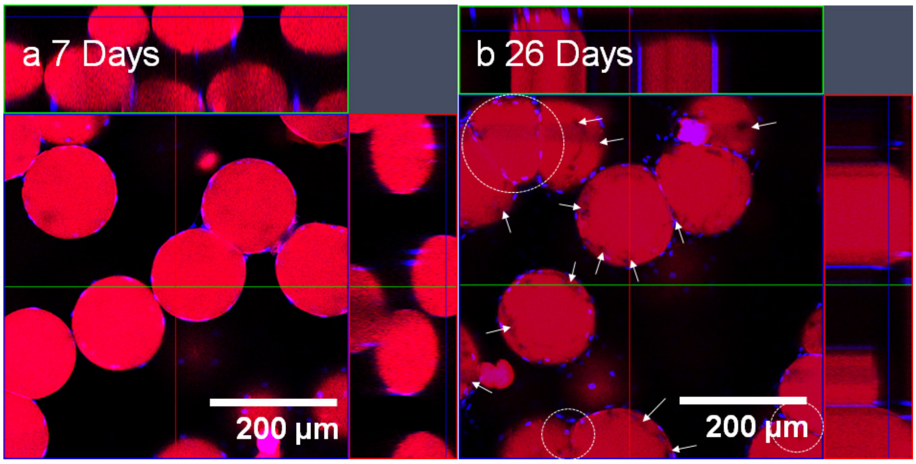

Figure S6. *In vitro* study of CLSM images of AMH with hADSC in a) 7 and b) 26 days. AMH was degraded (white arrow) by hADSC and became deformation (white circle).

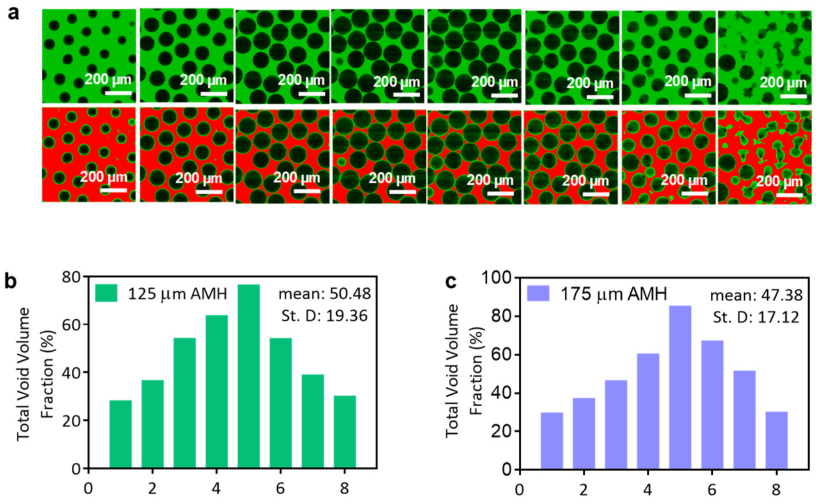

Figure S7. a) Fluorescent image process diagram from original image to labelled pores. b-c) Void volume fraction analysis in different size of building blocks.

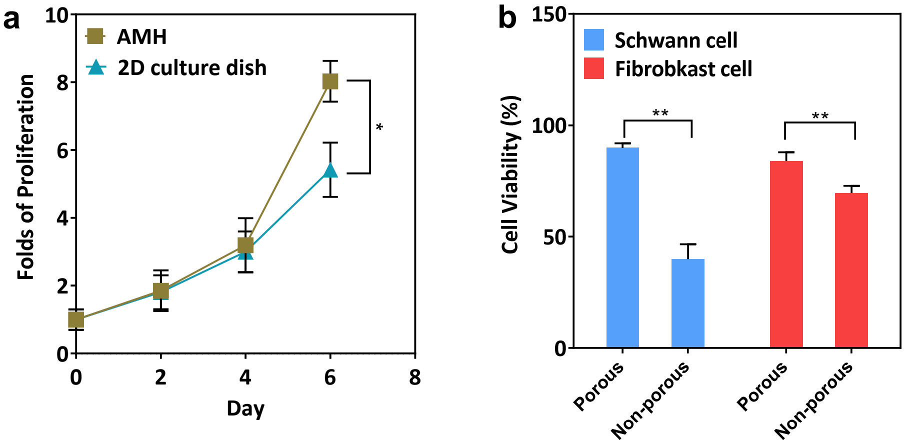

Figure S8. a) The proliferations of SCs on AMH and a 2D cell culture dish (control) during the period of 6 days. b) Cell viability of survival SC and fibroblast cells in in AMH and non-porous gel for 24 h (n = 6, mean ± s.d., *$p < 0.05$, **$p < 0.01$, *t*-test).

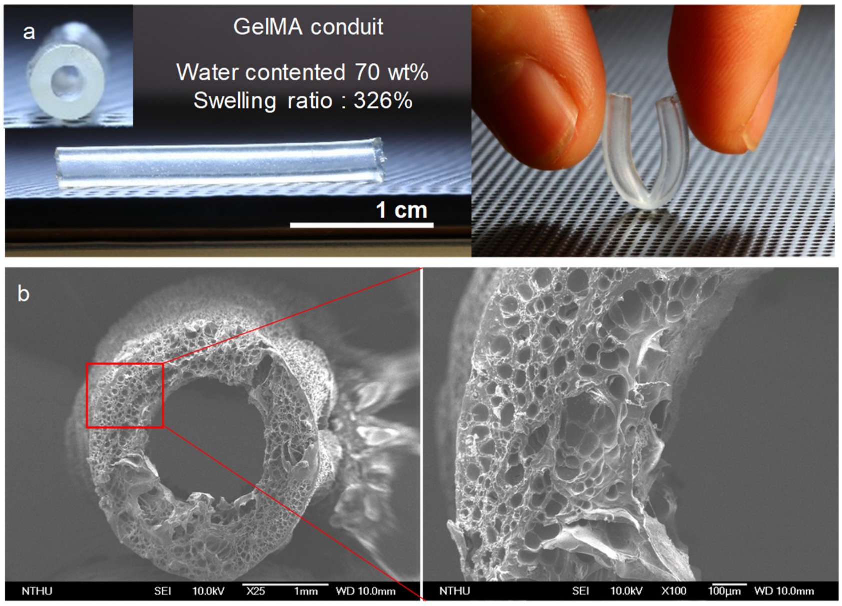

Figure S10. a) The conduit for in vivo study made by GelMA through molding and freeze-drying process. The conduit exhibited the swelling ratio of 326% with smooth surface and flexible property. b) The SEM images of conduit after drying process. After drying, a porous structure with a mean pore size of 50 μm in the conduit.

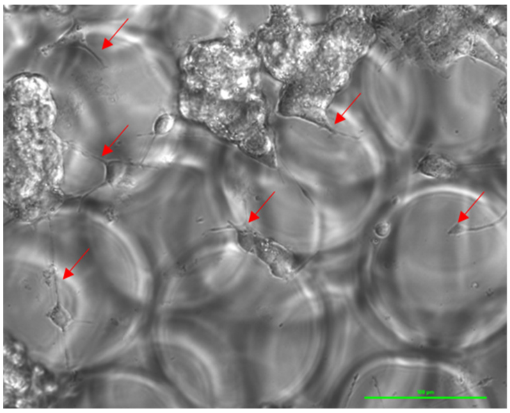

Figure S11. Released NGF from NGF-G-CL-beads stimulated the axon outgrowth of PC12 after 14 days.

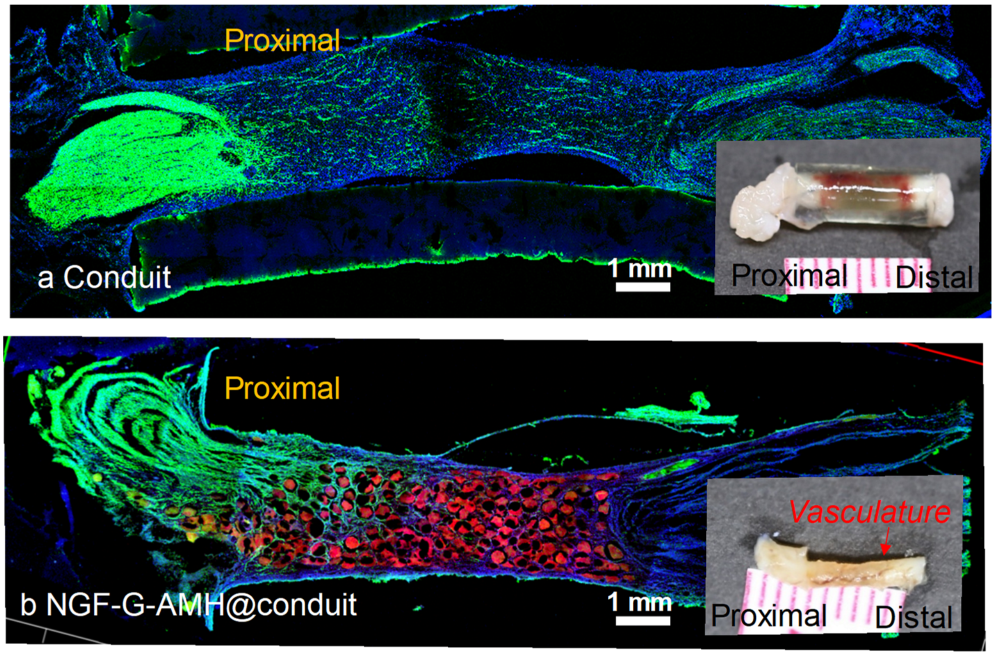

Figure S12. *In vivo* study of immunohistochemistry images of sciatic nerve defects of regenerated nerve tissues harvested from different sample groups: a) conduit and b) NGF-G-AMH@conduit at 7 days post-surgery. The expression of axons (green) was analyzed by immunofluorescence; AMH (red) and nucleic (blue).

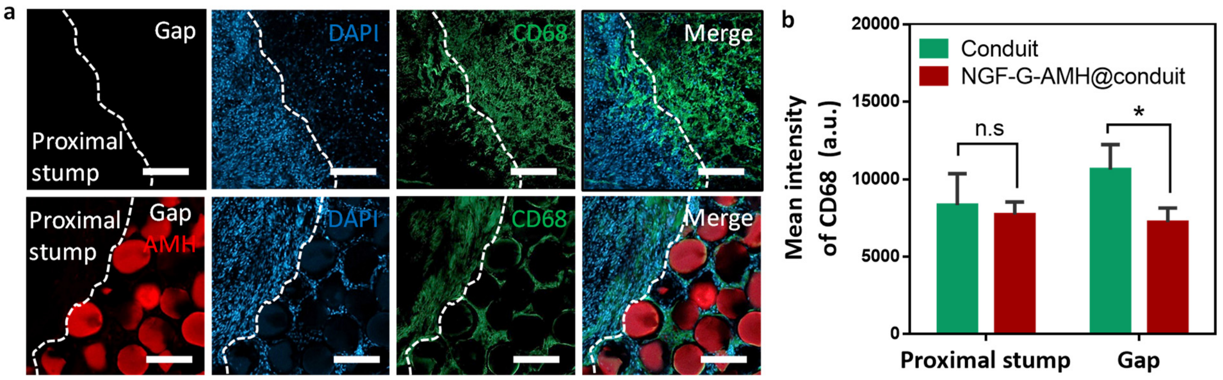

Figure S13. The proximal nerve segments at lesion sites were collected and quantification of immune response at 4 days post-surgery. a) CLSM images of nerve repair of conduit and NGF-G-AMH@conduit after 4 days post-surgery. The scale bar is 200 μm. b) Mean intensity of CD68-positive cells in conduit and NGF-G-AMH@conduit (n = 5, mean ± s.d., *p < 0.05, *t*-test).

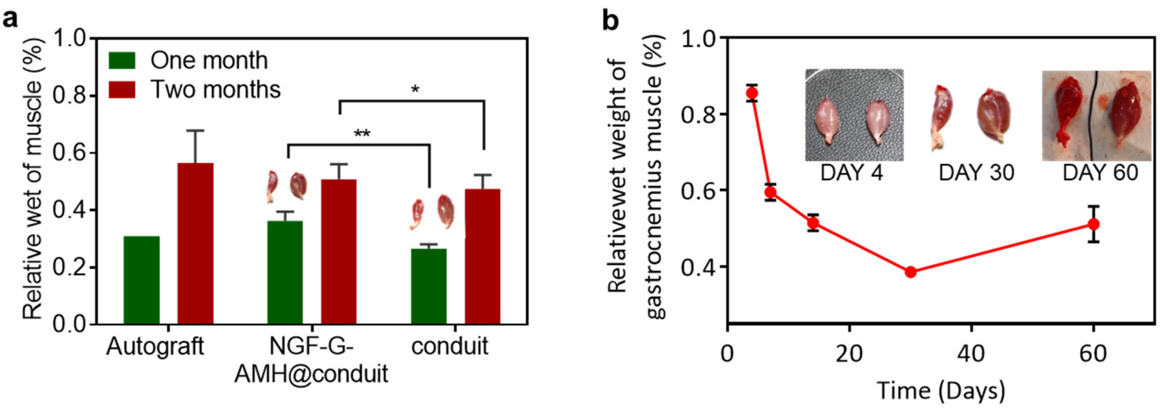

Figure S14. Measurement of gastrocnemius muscle wet weight. a) Relative gastrocnemius muscle wet weights of the injured limbs to the contralateral limbs after one month and two months of the implantation of autograft, NGF-G-AMH@conduit and conduit (n = 5, **p* < 0.05, ***p* < 0.01, one-way ANOVA followed by Tukey's post hoc analysis). b) The relative gastrocnemius muscle wet weight after implanting NGF-G-AMH@conduit at various times (n = 5, mean ± s.d.).

Movie S1. Three-dimensional reconstructed confocal microscopic image of AMH.
Movie S2. Mechanical property and stability of AMH in water.

[S1] H. Shirahama, B. H. Lee, L. P. Tan, N. J. Cho, *Sci. Rep.* **2016,** *6,* 31036.
[S2] J. W. Nichol, S. T. Koshy, H. Bae, C. M. Hwang, S. Yamanlar, A. Khademhosseini, *Biomaterials* **2010,** *31,* 5536.
[S3] J. R. Bain, S. E. Mackinnon, A. R. Hudson, R. E. Falk, J. A. Falk, D. A. Hunter, *Plast. Reconstr. Surg.* **1988,** *82,* 447.
